# Modified recombinant collagen-peptide/gallic acid grafted chitosan composites with antibacterial and antioxidant property for wound dressing: a preliminary study

**DOI:** 10.3389/fimmu.2025.1675212

**Published:** 2025-09-15

**Authors:** Yu Chen, Wenjie Wen, Jie Wang, Shiting Huang, Qianqian Ren, Jiayao Cai, Songlin Zhou, Rui Li, Dandan Ren, Liaotian Peng, Chao Deng, Jue Zhang

**Affiliations:** ^1^ Anhui Province Engineering Research Center for Dental Materials and Application, School of Stomatology, Wannan Medical College, Wuhu, China; ^2^ School of Laboratory Medicine, Wannan Medical College, Wuhu, China

**Keywords:** recombinant collagen-peptide, antibacterial property, wound repair, RHC based hydrogel, antioxidants

## Abstract

Chronic wounds pose a persistent clinical challenge, primarily due to prolonged bacterial infections. The development of natural antibacterial dressings offers a promising strategy for their effective management. In this study, recombinant human collagen methacrylamide (RHCMA) and gallic acid-grafted chitosan (CSGA) were synthesized and subsequently crosslinked via UV irradiation to form a composite hydrogel (RHCMA-CSGA). The composition and structure of the hydrogel were systematically characterized. Its cytocompatibility and antibacterial properties were also evaluated. The results demonstrated that the hydrogel exhibited excellent biocompatibility and strong antibacterial activity. These findings suggest that the RHCMA-CSGA hydrogel holds great potential as a therapeutic dressing for chronic wound healing.

## 1 Introduction

The skin acts as the body’s primary physical and immunological barrier against external environmental threats (1–3). Under pathological conditions such as diabetes mellitus, chronic venous insufficiency, prolonged pressure, severe infections, or impaired vasculature, the skin’s regenerative capacity may be compromised, leading to the formation of chronic, non-healing wounds (4, 5). These wounds not only cause severe discomfort to patients but also present a major clinical burden. Therefore, the development of effective treatments and advanced wound dressings remains a key focus in chronic wound management (6).

Research indicates that antioxidant and antibacterial therapies play a crucial role in the management of chronic wounds. Currently, clinical interventions predominantly depend on antibiotics and related pharmaceutical agents; however, this approach may result in various complications, including the development of bacterial resistance (7). Utilizing natural extract-based hydrogels to deliver antioxidant and antibacterial properties represents a promising strategy to address these challenges. Consequently, the development of multifunctional hydrogels exhibiting both antioxidant and antibacterial capabilities has emerged as a significant area of focus in contemporary biomedical research (8).

Collagen, a principal component of the extracellular matrix, exhibits excellent biocompatibility and plays a vital role in tissue regeneration (9). Recombinant human collagen peptides (RHC) are engineered biomimetic analogues of native collagen that actively participate in embryonic development and early-stage wound healing (10). RHC has emerged as a well-defined, reliable, and predictable biomaterial with favorable biological properties (11). Due to its low immunogenicity and good biocompatibility, RHC is considered a promising candidate for tissue engineering. However, its mechanical performance and functional properties still require optimization (12, 13).

Traditionally, RHC-based materials are crosslinked using chemical agents such as glutaraldehyde or EDC-NHS, which may raise cytotoxicity concerns (14). To circumvent this, RHC can be modified with methacrylamide groups, rendering it photo-crosslinkable and suitable for UV-induced gelation (15). The resulting RHCMA hydrogels retain biocompatibility and exhibit minimal cytotoxicity, making them particularly suitable for wound healing applications (16, 17).

Antibacterial activity is a critical functional property for wound dressings, especially in the treatment of chronic wounds, which are often complicated by persistent infections (18–21). Chitosan (CS), a naturally derived polysaccharide, is well known for its biocompatibility, biodegradability, and intrinsic antimicrobial activity, which supports its widespread use in biomedical applications (22, 23). Grafting gallic acid (GA; 3,4,5-trihydroxybenzoic acid) onto chitosan has been reported to enhance its antimicrobial and antioxidant properties (24–26).

In this study, RHCMA was employed as the structural backbone, while CSGA was incorporated into the hydrogel matrix via hydrogen bonding interactions (27). The objective was to develop and characterize a novel composite hydrogel for chronic wound treatment. The hydrogel was characterized using nuclear magnetic resonance (NMR), Fourier-transform infrared spectroscopy (FTIR), scanning electron microscopy (SEM), and rheological analysis. Additionally, its cytocompatibility and antibacterial efficacy were assessed. This composite hydrogel offers a promising platform for application in chronic wound repair.

## 2 Materials and methods

### 2.1 Materials

Recombinant humanized type III collagen (RHC, Mw = 25.564 kDa) was obtained from Jland Biotech Co., Ltd. (Jiangsu, China). Chitosan (CS, Mw = 50–190 kDa; degree of deacetylation (DD) = 82%) was purchased from Sigma-Aldrich (St. Louis, MO, USA). Gallic acid (GA), N-hydroxysuccinimide (NHS), 1-(3-dimethylaminopropyl)-3-ethylcarbodiimide hydrochloride (EDC), and methacrylic anhydride (MA) were all purchased from Bide Pharmatech Ltd. (China). Deionized (DI) water was used for all experiments.

For cell culture, Dulbecco’s Modified Eagle Medium (DMEM) and penicillin/streptomycin were acquired from BioSharp Co., Ltd. (Hefei, China), and fetal bovine serum (FBS) was supplied by Zhejiang Tianhang Biotechnology Co., Ltd. (Zhejiang, China). The EdU assay kit was purchased from Elabscience Co., Ltd. (China), and the Calcein-AM/PI Cell Viability/Cytotoxicity Assay Kit was obtained from Beyotime Biotechnology Co., Ltd. (China).

#### 2.1.1 Synthesis of recombinant collagen methacrylamide

Recombinant type III collagen (1 g) was dissolved in 7 mL of Na_2_CO_3_–NaHCO_3_ buffer under constant stirring at room temperature. After complete dissolution, the solution was heated to 40 °C, followed by the dropwise addition of 0.1 mL methacrylic anhydride. The reaction was maintained with continuous stirring for 4 h. The resulting mixture was dialyzed against deionized water for 3 days (molecular weight cutoff: 3500 Da) and then lyophilized for further use (28).

#### 2.1.2 Synthesis of chitosan–gallic acid conjugate

The chitosan–gallic acid conjugate was synthesized via EDC/NHS-mediated coupling between chitosan and GA (29). Briefly, 1 g of chitosan was dissolved in 100 mL of 0.5% (v/v) acetic acid and stirred overnight. The pH of the solution was adjusted to 5.0 using sodium hydroxide. GA (1.054 g, equimolar to chitosan) was dissolved in 10 mL of ethanol and slowly added to the chitosan solution. EDC (1.304 g, 1.1 molar equivalents) and NHS (0.782 g, 1.1 molar equivalents), each dissolved in 10 mL DI water, were added dropwise to initiate the coupling reaction. The mixture was stirred under nitrogen at 25 °C for 12 h. The final product was dialyzed (MWCO: 3500 Da) against DI water for 3 days and then freeze-dried.

### 2.2 Preparation and characterization of RHCMA-CSGA and RHCMA-CS hydrogels

#### 2.2.1 Hydrogel preparation

Methacrylated RHC (1 g) was dissolved in 10 mL DI water containing 0.25% (w/v) LAP photoinitiator to prepare a 10% (w/v) RHCMA solution. CS and CSGA were each dissolved in 0.5% (v/v) acetic acid to obtain 1 mg/mL solutions. These were then mixed with the RHCMA solution at a volume ratio of 1:2. The mixtures were photo-crosslinked under visible light (405 nm) to form RHCMA-CSGA and RHCMA-CS hydrogels, followed by additional UV irradiation (20 s) to ensure complete crosslinking, after which the hydrogels were subjected to further experiments.

#### 2.2.2 Physicochemical characterization

The chemical structures of RHCMA and CSGA were analyzed using Fourier-transform infrared spectroscopy (FTIR, Thermo Fisher Nicolet 380) and nuclear magnetic resonance (NMR, Bruker, Switzerland).The degree of substitution (%) of the CS-GA conjugate was determined from the peak area of the aromatic protons of GA with respect to that of the acetyl protons of CS. Functional group characterization of the hydrogels was performed via FTIR. Crystallinity was assessed by wide-angle X-ray diffraction (XRD, Rigaku SmartLab) within the range of 10°–80°. Rheological behavior was measured using a rheometer (Anton Paar, MCR 702e). Morphological analysis of freeze-dried hydrogel samples was conducted via scanning electron microscopy (SEM, Hitachi S-4800).

### 2.3 Antibacterial assay

The antibacterial activity of the hydrogels was tested against *Escherichia coli* (BW25113) and methicillin-resistant *Staphylococcus aureus* (MRSA, ATCC29213), obtained from the State Key Laboratory of Bio-Control and the Guangdong Key Laboratory of Pharmaceutical Functional Genes. Bacterial cultures were incubated at 37 °C for 24 h and diluted 100-fold in fresh LB medium to achieve a final concentration of 10^6^ CFU/mL. Then, 1 mL of the bacterial suspension was incubated with hydrogel samples (1 mg/mL) in 1.5 mL centrifuge tubes at 37 °C for 24 h. After incubation, bacterial suspensions were serially diluted in sterile PBS, and 100 μL of each dilution was plated on LB agar and incubated at 37 °C for 18 h. Colony-forming units (CFUs) were counted, and representative images were captured for quantitative analysis.

### 2.4 *In vitro* antioxidant performance

#### 2.4.1 ABTS radical scavenging assay

The ABTS radical scavenging capacity was measured following a reported method (30). Briefly, different volumes of sample solutions were added into a 96-well microplate preloaded with 200 µL ABTS solution. The stock ABTS solution was diluted with PBS to achieve an initial absorbance of ~1.0 at 734 nm after sample addition. The plate was incubated at room temperature for 30 min, and absorbance was then recorded at 734 nm using a microplate reader. The decrease in absorbance was positively correlated with antioxidant activity.

#### 2.4.2 DPPH radical scavenging assay

The DPPH radical scavenging ability was evaluated according to previous methods (30). Different volumes of sample solutions were added into a 96-well microplate, followed by 200 µL of 0.2 mM DPPH methanol solution in each well. The plate was incubated in the dark at room temperature for 30 min. Absorbance was measured at 517 nm using a microplate reader. The reduction in absorbance reflected the scavenging ability of samples toward DPPH radicals.

### 2.5 *In vitro* cytocompatibility evaluation

#### 2.5.1 Cell culture and extract preparation

L929 mouse fibroblasts (Wuhan Pricella Biotechnology Co., Ltd.) were cultured in DMEM supplemented with 10% FBS and 1% penicillin–streptomycin at 37 °C in a 5% CO_2_ humidified atmosphere. RHCMA-CSGA and RHCMA-CS hydrogels were dispersed in DMEM at concentrations of 0.2, 0.5, and 1.0 mg/mL. These suspensions were shaken at 200 rpm at 37 °C for 24 h to prepare extract solutions. The extracts were then filtered through 0.22 μm syringe filters and supplemented with 10% FBS and 1% antibiotics prior to use.

#### 2.5.2 Cytotoxicity assessment

Cytotoxicity and cell proliferation were assessed using CCK-8 assays as described previously (30). L929 cells were seeded in 24-well plates at a density of 5 × 10^4^ cells/well and incubated overnight. The medium was then replaced with sample extracts, and cells were incubated for 24 h. After removing the extracts, 300 μL of fresh medium containing 10% CCK-8 solution was added, and the plates were incubated for 2 h. Absorbance at 450 nm was recorded using a microplate reader (Cytation 3, BioTek, USA).

Live/dead staining was conducted using Calcein-AM (live cells) and PI (dead cells) after co-culture with 1.0 mg/mL sample extracts. Following a 24 h incubation, cells were harvested by centrifugation at 800 rpm, resuspended in staining solution (0.4% Calcein-AM and 0.5% PI), and incubated at 37 °C for 30 min. Fluorescence images were captured using a fluorescence microscope (Olympus IX73, Japan), and ImageJ software was used for live/dead cell ratio analysis.

#### 2.5.3 EdU-based cell proliferation assay

Cell proliferation was evaluated using an EdU assay kit (Elabscience, China) as per the manufacturer’s instructions and previous literature (30). L929 cells were seeded at 2 × 10^4^ cells/well in 24-well plates and incubated for 24 h. EdU labeling was performed using EdU working solution, followed by fixation with 4% paraformaldehyde and permeabilization with 0.2% Triton X-100. EdU reaction solution was added to stain the newly synthesized DNA, and nuclei were counterstained with DAPI. Fluorescence images were acquired using a fluorescence microscope (Olympus IX73, Japan), and EdU-positive cells were quantified using ImageJ.

## 3 Results and discussion

**Figure 1:**
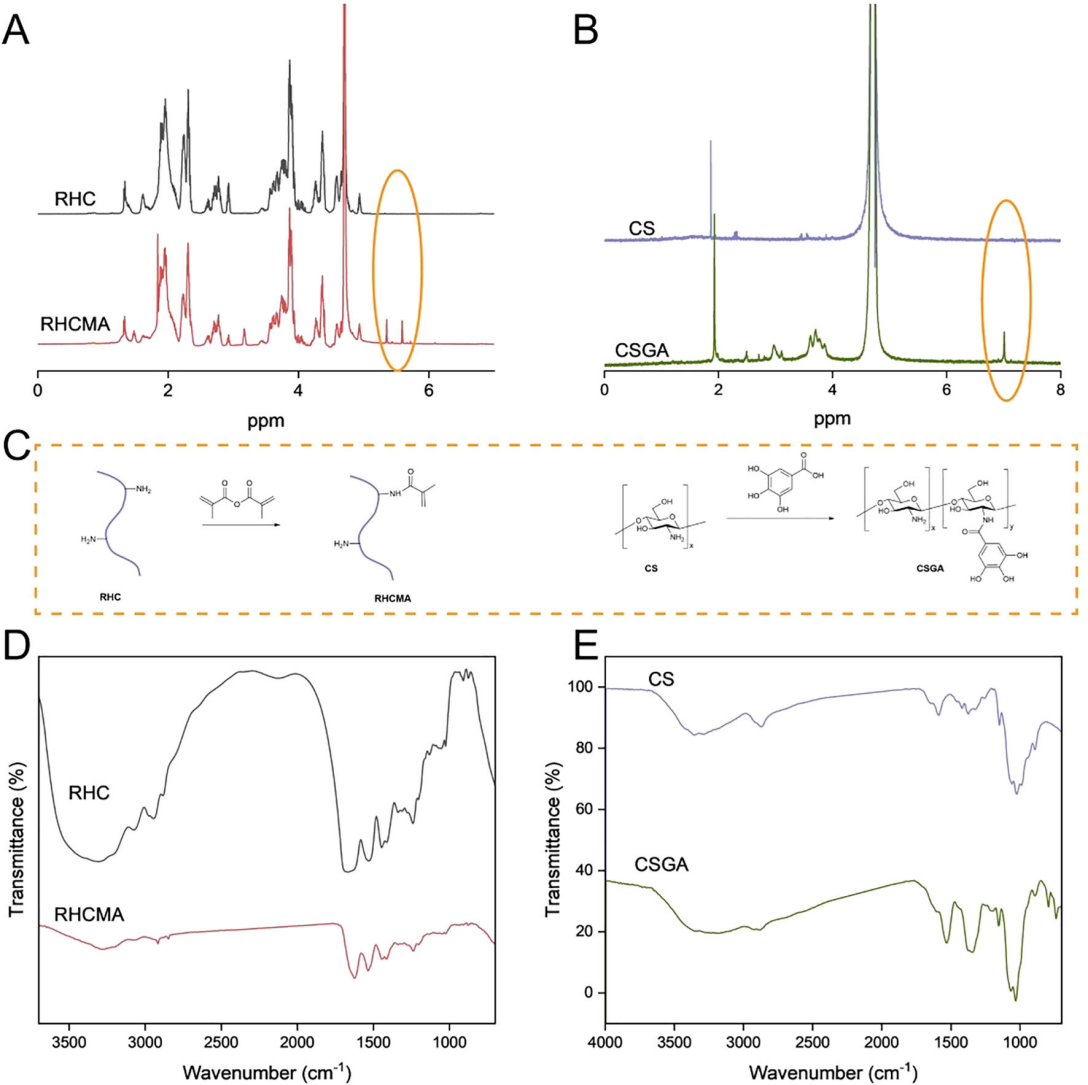
The structure and synthesized process illustration of RHCMA and CSGA **(C)**; The NMR **(A, B)** and FTIR **(D, E)** spectras of RHC, RHCMA, CS and CSGA.

To fabricate the RHCMA-CSGA hydrogel, recombinant human type III collagen methacrylamide (RHCMA) and chitosan–gallic acid conjugate (CSGA) were synthesized as monomer precursors. The synthetic schemes are illustrated in **Figure 1C**. The chemical structures of RHCMA and CSGA were confirmed via ^^1^H NMR spectroscopy (**Figures 1A, B**). Specifically, RHCMA exhibited characteristic proton signals at 5.4 and 5.7 ppm, corresponding to the vinyl protons of the methacrylate group (28). For CSGA, a multiplet in the 6.9–7.1 ppm range was observed, which was attributed to the aromatic protons of the gallate moiety (29), confirming successful grafting of gallic acid onto the chitosan backbone. The degree of substitution (%) of the CS-GA conjugate was 30%.

As depicted in **Figure 1C**, RHCMA and CSGA were synthesized via a two-step chemical modification. RHC was first functionalized with methacrylic anhydride to introduce photocrosslinkable methacrylate groups. In parallel, chitosan was covalently modified with gallic acid via an EDC/NHS-mediated coupling reaction, endowing the conjugate with phenolic hydroxyl groups that impart antibacterial and antioxidant properties.

Further structural confirmation was obtained via FTIR spectroscopy (**Figures 1D, E**). RHCMA displayed absorption peaks at 1627 cm^-1^ (amide I band, C=O stretching), 1542 cm^-1^ (amide II band, N–H bending), and 1053 cm^-1^ (C=C–H in-plane bending), consistent with the successful introduction of methacrylate moieties (28). In the spectrum of native CS, the peak at 1584 cm^-1^ (N–H bending) was absent in the CSGA spectrum. GA exhibited characteristic aromatic C=C stretching vibrations between 1450–1600 cm^-1^. The disappearance of the 1584 cm^-1^ peak confirmed successful conjugation of GA to the amino groups of CS (29).

**Figure 2:**
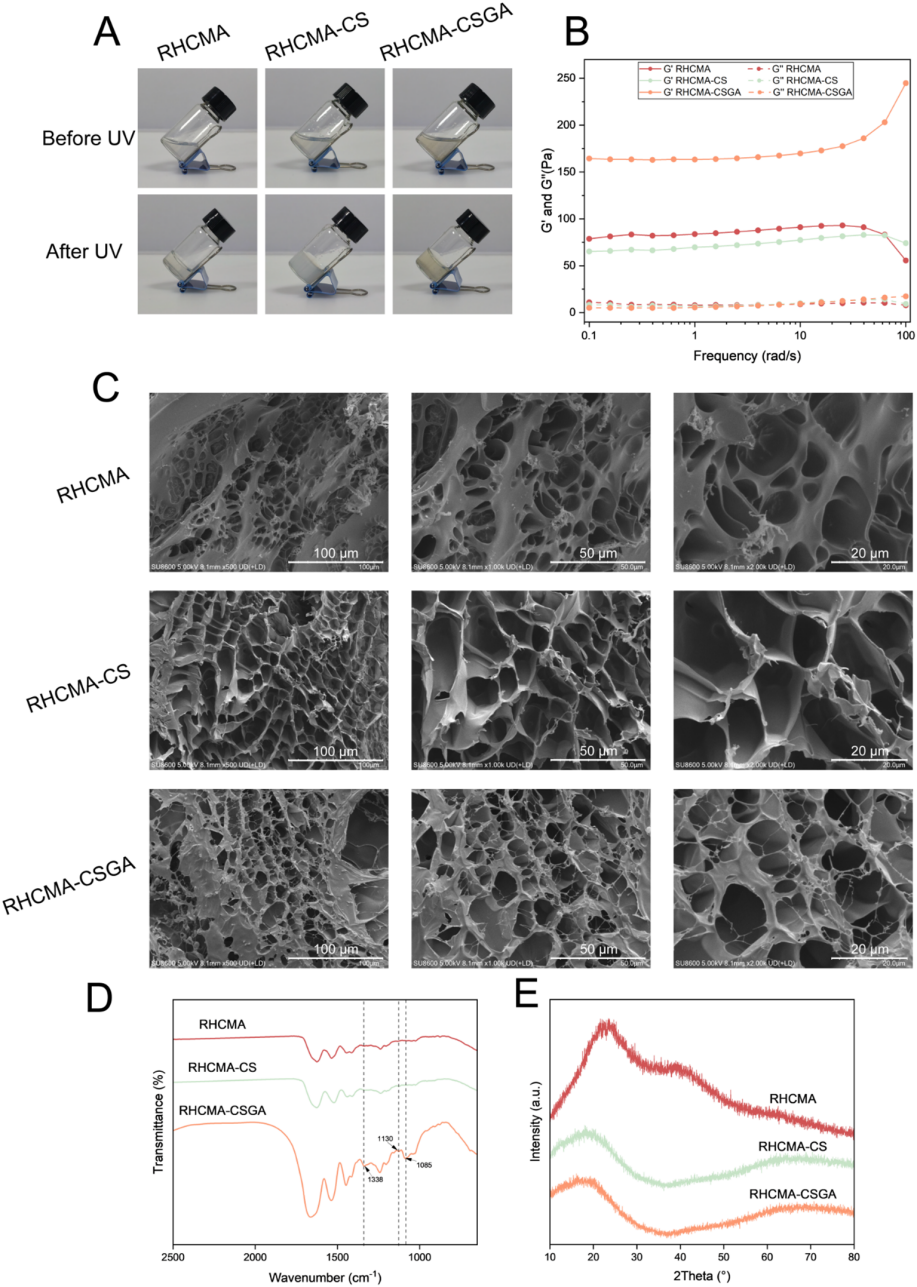
Characterizing of hydrogels. Photographs **(A)**, rheological properties **(B)**, SEM images **(C)**, FTIR spectras **(D)** and XRD patterns **(E)** of RHCMA, RHCMA-CS and RHCMA-CSGA.

To evaluate the photo-crosslinking efficiency of the different formulations (RHCMA, RHCMA-CS, and RHCMA-CSGA), the mixtures were exposed to 405 nm UV light for 15 seconds (**Figure 2A**). Oscillatory rheological analysis was conducted to assess their viscoelastic properties. As shown in **Figure 2B**, for all hydrogels, the storage modulus (G′) exceeded the loss modulus (G″) across the entire frequency range (0–100 rad/s), indicating stable gel networks and solid-like behavior.

SEM images of the freeze-dried hydrogels revealed distinct morphological differences (**Figure 2C**). The RHCMA hydrogel exhibited a porous structure typical of freeze-dried networks. The addition of CS led to a noticeable reduction in porosity, while CSGA incorporation further minimized the formation of freeze-dried pores. These observations, coupled with rheological data, suggest that while CS had minimal impact on network stiffness, CSGA significantly enhanced crosslinking density. This enhancement likely results from the phenolic hydroxyl groups in the gallic acid moiety forming extensive hydrogen bonding with the hydroxyl groups in RHC, thus reinforcing the hydrogel matrix.

The FTIR spectrum of the RHCMA-CSGA hydrogel (**Figure 2D**) exhibited a prominent absorption peak at 1085 cm^-1^, corresponding to C–O and C–C stretching vibrations, further validating the successful crosslinking of components. XRD analysis demonstrated broad amorphous humps spanning 10°–80°for all hydrogel samples, indicating their non-crystalline nature (**Figure 2E**).

**Figure 3:**
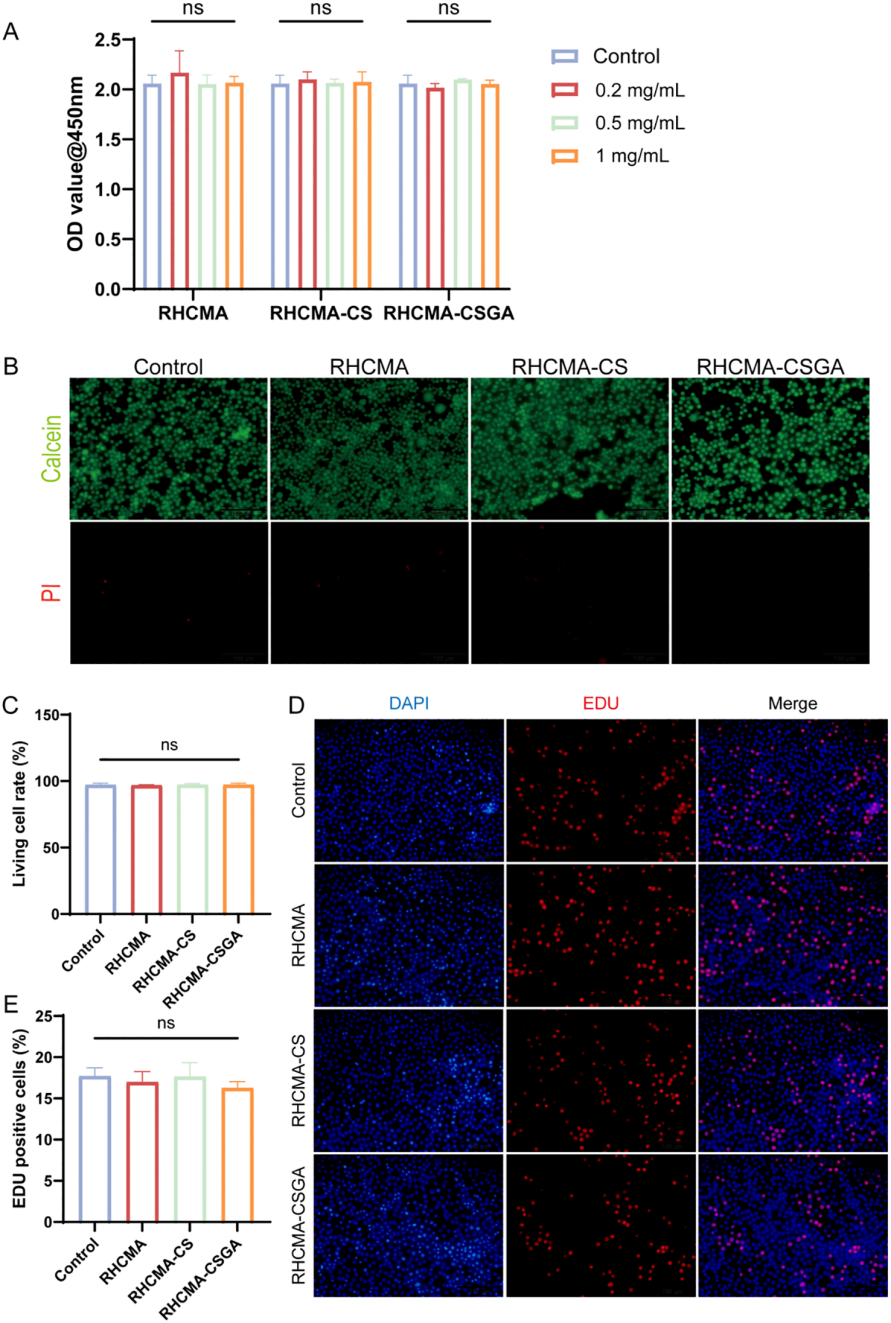
The cytotoxicity and EDU evaluation. **(A)** OD value at 450nm of L929 culture with extracts of different samples in 0.2, 0.5, and 1mg/mL. **(B)** Live and dead staining. Fluorescence imaging of calcein and PI staining of samples in extracts of 1 mg/mL. **(C)** Quantitative evaluation of live and dead staining. **(D)** Fluorescence imaging of DAPI and EDU staining of samples and **(E)** quantitative analysis in extracts of 1 mg/mL. “ns” means no significant.

The cytotoxicity and proliferation effects of RHCMA, RHCMA-CS, and RHCMA-CSGA were evaluated using L929 fibroblasts. Extracts of the samples were prepared at concentrations of 0.2 mg/mL, 0.5 mg/mL, and 1 mg/mL after 24-hour co-cultured with cells. At 0.2, 0.5 and 1 mg/mL, all groups showed no cytotoxicity (**Figure 3A**). Based on the overall performance, the 1 mg/mL was selected for further testing.

To further visualize cytotoxic effects, live/dead staining was performed after 24-hour co-culture of L929 cells with 1 mg/mL extracts (**Figure 3B**). No significant cytotoxicity was observed in any group compared to the control (**Figure 3C**). Additionally, an EdU proliferation assay was conducted to examine the effect of each group on L929 cell proliferation (**Figure 3D**). Results showed that none of the 1 mg/mL extracts promoted cell proliferation (**Figure 3E**). The hydrogels composited of RHCMA and CSGA derived from natural product modifications exhibited low toxicity.

**Figure 4:**
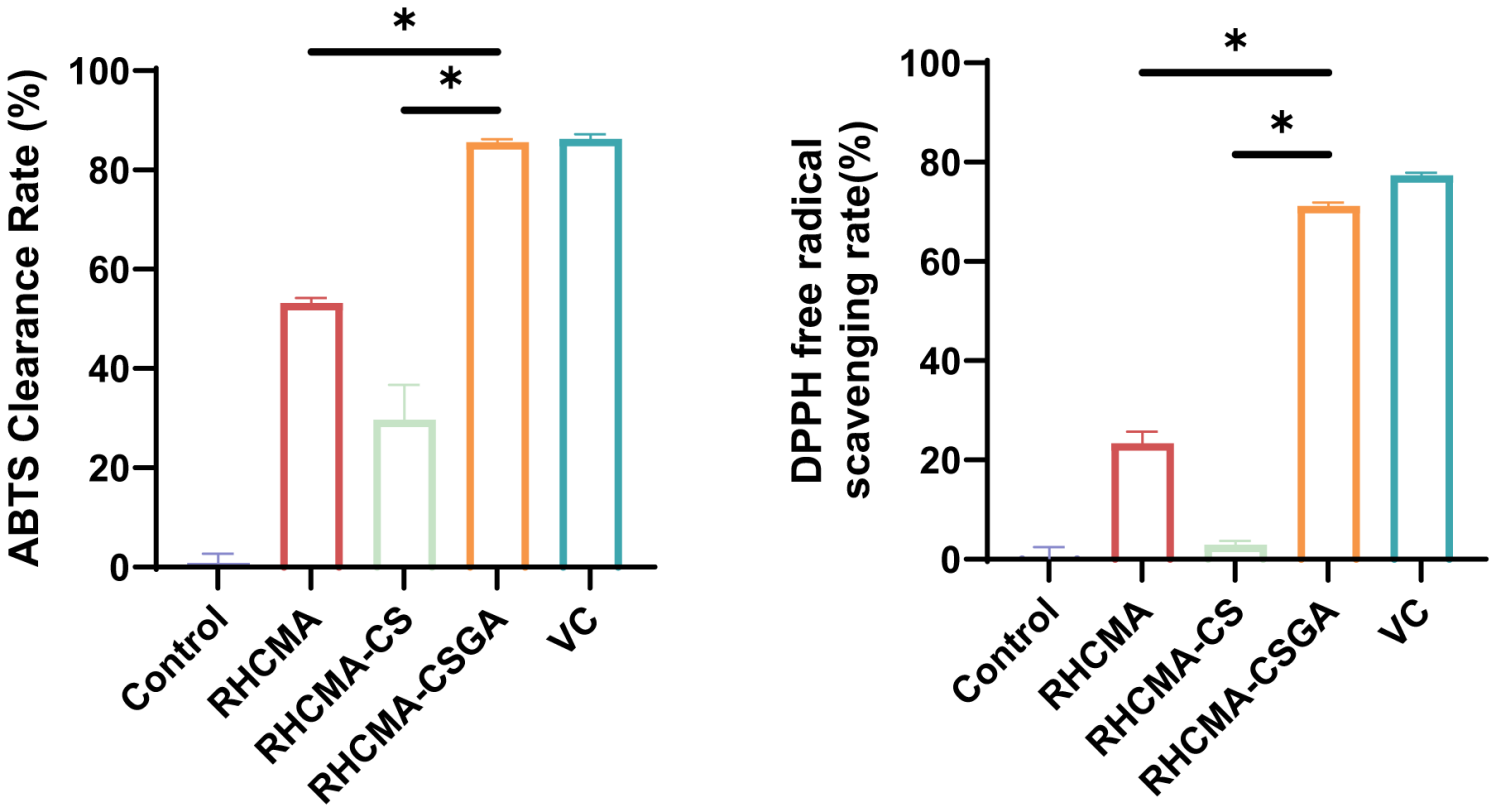
Evaluation of antioxidant properties of RHCMA, RHCMA-CS, and RHCMA-CSGA: ABTS radical generation assay and DPPH free radical-scavenging rate determination; “*” means p value<0.05.

The antioxidant properties were assessed using DPPH and ABTS assays, two widely accepted spectrophotometric methods for evaluating non-enzymatic radical-scavenging activity. As shown in **Figure 4**, RHCMA-CSGA exhibited significantly enhanced radical-quenching capacity compared to both RHCMA and RHCMA-CS. Among the tested samples, RHCMA-CS presented the lowest activity, whereas RHCMA-CSGA demonstrated the highest, with ABTS and DPPH scavenging values approaching those of ascorbic acid (vitamin C).

Phenolic hydroxyl groups are well recognized for their potent antioxidative activity, and their covalent incorporation into polymeric backbones is known to markedly improve radical scavenging performance. In this study, grafting phenolic hydroxyl moieties onto chitosan (CS) resulted in a pronounced enhancement of antioxidant activity. In contrast, the poor activity of RHCMA-CS is attributed to its insufficient phenolic hydroxyl content, which limits its ability to effectively participate in hydrogen atom transfer and single-electron transfer reactions essential for radical quenching and chain-breaking antioxidant mechanisms.

In the context of wound healing, antibacterial activity is essential for preventing infection and promoting tissue regeneration. While antibiotics remain the primary agents in clinical use, they are associated with rising concerns over antimicrobial resistance. Natural antibacterial components offer a safer and sustainable alternative. In this study, we enhanced the antimicrobial activity of CS by grafting it with gallic acid.

**Figure 5:**
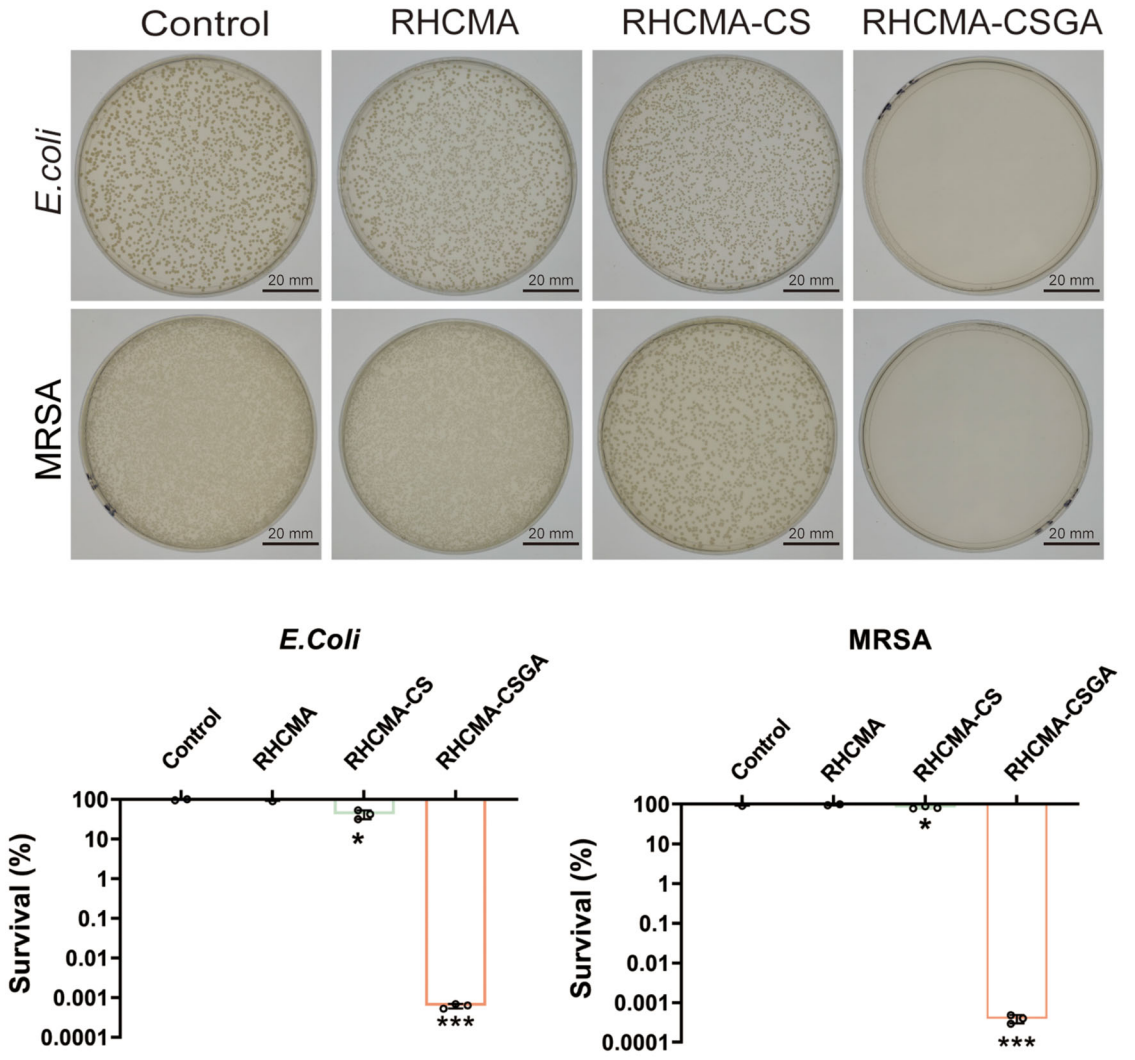
Antibacterial property evaluation against *E.Coli* and MRSA of RHCMA, RHCMA-CS and RHCMA-CSGA;”*” means compare with control group, P<0.05; “***”means compare with control group, P<0.001.

The antibacterial efficacy of the hydrogels was assessed by co-culturing them with Escherichia coli and methicillin-resistant Staphylococcus aureus (MRSA), followed by plate counting. As shown in **Figure 5**, RHCMA-CSGA exhibited potent antibacterial activity against both strains. RHCMA-CS demonstrated moderate inhibition of *E. coli* but was ineffective against MRSA. The RHCMA hydrogel alone showed no observable antibacterial activity compared to the control.

The observed antibacterial enhancement is attributed to the combined effects of chitosan’s intrinsic polycationic nature, which disrupts bacterial membranes, and the gallate moiety, known for its broad-spectrum antibacterial activity. Phenolic moieties are known to exert strong antimicrobial effects by generating oxidative stress through redox cycling and by directly interacting with bacterial membranes, thereby increasing membrane permeability and disrupting cellular integrity (31). Additionally, phenolic groups can chelate essential metal ions, further interfering with bacterial enzymatic functions and metabolic pathways (32).Beyond the individual contribution of GA, a synergistic effect between chitosan (CS) and GA likely accounts for the superior antibacterial activity of RHCMA-CSGA. Protonated amino groups on CS interact electrostatically with negatively charged bacterial cell walls, leading to increased membrane permeability. The incorporation of GA amplifies this disruption through phenolic-induced oxidative stress and further destabilization of the membrane (33). Consequently, the combined action of CS and GA provides a dual antibacterial mechanism, which explains the significantly enhanced efficacy of RHCMA-CSGA hydrogels relative to other formulations.

Importantly, the CSGA-containing hydrogel retained its antibacterial function even at relatively low concentrations, making it a promising candidate for chronic wound applications.

## 4 Conclusion

In this study, a novel composite hydrogel composed of methacrylated recombinant collagen (RHCMA) and gallic acid-grafted chitosan (CSGA) was successfully developed. The incorporation of CSGA significantly enhanced the gel stiffness and network density, as confirmed by rheological and morphological analyses. *In vitro* evaluations demonstrated that the RHCMA-CSGA hydrogel was biocompatible and exhibited low cytotoxicity. Although it did not promote fibroblast proliferation, it maintained excellent cellular compatibility. Moreover, the hydrogel exhibited strong antibacterial activity, particularly due to the incorporation of gallic acid, making it a promising candidate for the treatment of chronic wounds. These findings highlight the RHCMA-CSGA hydrogel’s potential as a multifunctional biomaterial for future wound care applications.

## Data Availability

The raw data supporting the conclusions of this article will be made available by the authors, without undue reservation.
